# Comparison of hook-wire and medical glue for CT-guided preoperative localization of pulmonary nodules

**DOI:** 10.3389/fonc.2022.922573

**Published:** 2022-08-08

**Authors:** Huijun Zhang, Ying Li, Xiaofeng Chen, Zelai He

**Affiliations:** ^1^ Department of Cardiothoracic Surgery, Huashan Hospital, Fudan University, Shanghai, China; ^2^ Department of Thoracic Surgery, Shanghai Pulmonary Hospital, School of Medicine, Tongji University, Yangpu, Shanghai, China; ^3^ Department of Radiation Oncology, the First Affiliated Hospital of Bengbu Medical College, Bengbu, China

**Keywords:** pulmonary nodules, hook-wire, medical glue, preoperative localization, video-assisted thoracic surgery (VATS)

## Abstract

**Background:**

Preoperative localization is challenging due to the small diameter of pulmonary nodules or their deep location in the lung parenchyma during VATS surgery. The purpose of this study was to compare the efficacy and safety of both hook-wire and medical glue for pre-operative localization of pulmonary nodules.

**Methods:**

In the current study, 158 patients were retrospectively analyzed (January 2019 and January 2020). The patients underwent hook-wire or medical glue for pre-operative localization of pulmonary nodules. Among them, 74 patients in the hook-wire group and 84 patients in the medical glue group underwent VATS anatomic segmentectomy or wedge resection after localization of pulmonary nodules. Pre-operative localization data from all patients were compiled. Moreover, the efficacy and safety of the two methods were evaluated according to localization success rates and localization-related complications.

**Results:**

The success rate of localization in the medical glue group was 100% while 97.3% in the hook-wire group. After localization of the pulmonary nodules, the incidence of minor pneumothorax in the medical glue group (11.9%) was lower than that in the hook-wire group (37.8%) (*p*=0.01). The incidence of mild pulmonary parenchymal hemorrhage in the medical glue group (13.1%) was also lower than that in the hook-wire group (24.3%) (p=0.000). The mean time from the completion of localization to the start of surgery was also longer in the medical glue group than in the hook-wire group (p=0.000). The mean visual analog scale (VAS) scores after localization were higher in the hook-wire group than in the medical glue group (*p*=0.02). In both groups, parenchymal hemorrhage was significantly associated with the needle length in hook-wire localization and the depth of the medical glue in the lung parenchyma (*p* = 0.009 and 0.001, respectively).

**Conclusion:**

These two localization methods are safe and effective in pre-operative pulmonary nodule localization. The medical glue localization method had a lower risk of complications, a higher localization success rate, less pain after localization and more flexibility in the arrangement of operation time.

## Introduction

Low-dose computed tomography (CT) has been widely used in early-stage lung cancer screening and can reduce lung cancer mortality by 20% (1). Therefore, the pathological detection of pulmonary nodules is particularly important. Compared to conventional procedures (transbronchial biopsy or CT-guided fine-needle aspiration biopsy),video-assisted thoracic surgery (VATS) for resection of pulmonary nodules yields more accurate pathological results and may also be curative (2, 3). However, most pulmonary nodules remain invisible or cannot be palpated during VATS due to their small diameter or deep location in the lung parenchyma. This results in a 54% rate of conversion from minimally invasive surgery to thoracotomy (4–6). Therefore, accurate localization of pulmonary nodules prior to surgical removal is crucial.

Several methods have been reported for pre-operative localization of pulmonary nodules, including intraoperative ultrasound (7, 8), hook-wire localization (9, 10), medical glue (11), micro-coil (12), methylene blue (13), indocyanine green (14), or radionuclides (15), some with 100% localization success rate (16). Pneumothorax or parenchymal hemorrhage are common complications during pre-operative localization, while fatal consequences are rarely reported (17, 18). There are not many studies on the effectiveness and safety of medical glue and hook-wire localization in localization technologies. In this study, we compared the above two methods of preoperative localization of pulmonary nodules and evaluated their efficacy and safety.

## Methods

### Study population

A retrospective analysis was performed for 158 patients with pulmonary nodules who underwent VATS resection with CT-guided pre-operative localization between January 2019 and January 2020. All patients received either CT-guided hook-wire or medical glue for localization. Among them, 74 patients had hook-wire localization, while the remaining 84 patients underwent medical glue localization. Subsequently, both groups had VATS anatomic segmentectomy or wedge resection. Before surgery, each patient had a discussion with a thoracic surgeon and a radiologist. The study was approved by the institution’s ethical review board, and all patients signed written informed consent. Inclusion criteria: Preoperative chest CT scan showed suspected malignant pulmonary nodules (≤30 mm) with the age of the patient being older than 18 years old. Exclusion criteria included: Pregnancy or lactation, severe coagulation disorders, severe cardiopulmonary dysfunction, severe infectious diseases, advanced tumors, and other patients who were not suitable for surgery. However, the operating doctor’s preferences determined the approach used to localize the lung nodules.

### Preoperative localization

The hook-wire localization needle was a double barb wire system made up of a 20-gauge breast puncture needle (10.7 cm long) and a BARD DUALOK trocar (Bard Peripheral Vascular, USA). The sleeve has a printed mark every 10 mm along the outside of the needle bar. First, a low-dose CT scan (120kV, 180mA, pitch 1.375, speed 55mm/0.5s, and reconstructed axial slice 2.5mm/2mm) was performed to confirm the precise localization of pulmonary nodules. Then, thoracic surgeons and radiologists performed a comprehensive assessment, paying particular attention to avoiding important structures (such as large blood vessels, interlobar pleura, scapula, and female breast tissue). The shortest and best puncture path was determined and the puncture point on the skin was recorded. After local anesthesia, the double-barb wire system was gradually inserted into the lung parenchyma under CT guidance, and the double-barb tip was deployed as close to the nodule as possible. After removal of the device, the needle bar remaining outside the patient’s body could be clipped off at the skin surface and covered with sterile gauze. In the medical glue group, we also used the hook-wire localization needle. When the localization needle tip reaches the appropriate area around the pulmonary nodules, the double-barb needle was pulled out, and then 0.2ml of medical-biological glue was injected as quickly as possible through the trocar before the trocar was withdrawn. The distance between the glue and the pulmonary nodules was controlled at about 1cm. To validate the localization effect and rule out problems like pneumothorax or pulmonary parenchymal hemorrhage, a CT scan was conducted after the first two procedures had been tried and shown to be effective. Concurrently, pain scores were measured using a visual analog scale (VAS) score.

### VATS surgery

**Figure 1:**
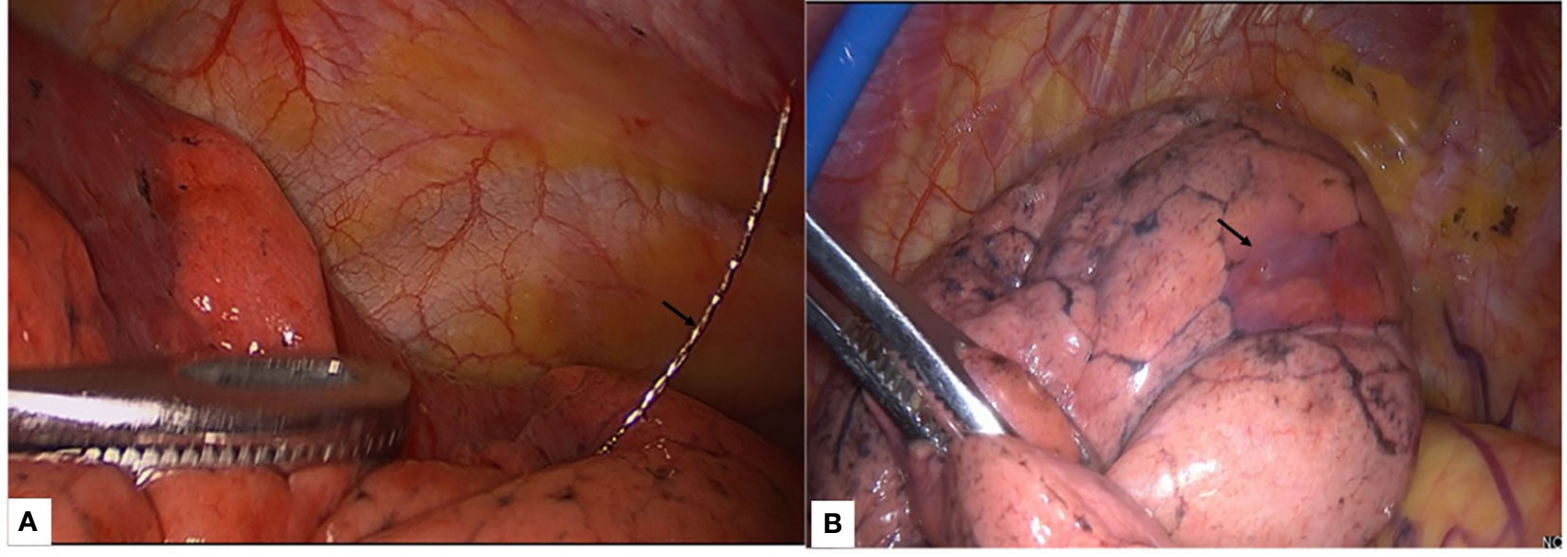
The localization of hookwire and medical glue in VATS surgery. **(A)** Hookwire needle (black arrow) located in the left lower lobe. **(B)** The medical glue (black arrow) is located in the upper lobe of the left lung, and the solid medical glue in the lung can be palpated with the fingers or determined by the needle puncture sites on the visceral pleura.

Under general anesthesia, VATS surgery was used to treat the hook-wire and medical glue groups. Surgical resection was performed using a standardized two-incision; placing the thoracoscopic port in the seventh intercostal space in the midaxillary line and the operative port in the fourth intercostal space in the anterior axillary line. During the operation, in the hook-wire group, the location of the pulmonary nodule was determined by the position of the hook-wire needle (**Figure 1A**). the medical glue group required the surgeon to determine the localization of the pulmonary nodule by physically touching the position of the medical glue with a finger (**Figure 1B**). The locations of the needle punctures on the visceral pleura were used to pinpoint the position of the medicinal adhesive in cases when it cannot be touched. Either wedge resection or anatomic segmentectomy was employed in all surgical techniques. To prevent the spread of malignant tumors, all surgical specimens were extracted in protective bags. A 28F chest tube was then inserted after the pleural cavity had been flushed with saline solution after surgery. Paraffin pathology reports were obtained for all patients post-operatively.

### Statistical analysis

Descriptive statistics for continuous variables are presented as mean ± SD. Continuous variables were analyzed using Student’s t-test. However, categorical variables were compared using the Chi-square test or Fisher’s exact test. *P* values less than 0.05 were considered statistically significant. All statistical analyses were performed using spss26.0 software.

## Results

### No statistical difference in the variables between the two groups

A total of 74 patients were assessed in the hook-wire group; including 38 males and 36 females. The average age was 51 ± 11.56 years(range: 27-82 years). The mean diameter of pulmonary nodules was 9.4 ± 3.0 mm (range: 6-15 mm). The average distance between the pulmonary nodules and the pleural surface was 20.6 ± 13.8 mm (range: 3-85 mm), and the average length of the hook-wire in the lung parenchyma was 29.4 ± 13.3 mm (range: 10-80.00 mm). There were 84 patients treated with medical glue; including 35 males and 49 females. The average age was 48.93 ± 14.04 years(range: 23-79 years). The mean diameter of the pulmonary nodules on CT was 10.04 mm (range: 4-25 mm). The mean distance of the pulmonary nodules from the pleural surface was 18.0 ± 12.3 mm (range: 3-88.00 mm) and the average distance from the glue to the pleural surface was 18.5 ± 12.9 mm (range: 4.10-96.40 mm). Moreover, the average time required for localization was 15.0 ± 3.83 min (range: 8-24 min). The above factors did not significantly vary between the two patient groups.

Within the hook-wire group, localization was in the right upper lobe in 30 patients (40.54%), in the middle lobe in 7 patients (9.46%), in the right lower lobe in 16 patients (21.62%), in the left upper lobe in 14 patients (18.92%), and in the left lower lobe in 7 patients (9.46%). In the medical glue group, 29 patients (34.52%) showed localization in the right upper lobe, 3 patients (3.57%) in the middle lobe, 27 patients (32.14%) in the right lower lobe, 19 patients (22.62%) in the left upper lobe, and 6 patients (7.14%) in the left lower lobe. There was no statistical difference between the two groups (p=0.325).

Pathological examination of the hook-wire group showed 3 cases (4.05%) of chronic inflammation, 3 cases (4.05%) of dysplasia, 3 cases (4.05%) of adenocarcinoma in situ, 46 cases (62.16%) of minimally invasive adenocarcinoma, and 19 cases (25.68%) of invasive adenocarcinoma. Within the medical glue group, pathological examination showed 10 cases (11.90%) of chronic inflammation, 4 cases (4.76%) of atypical hyperplasia, 8 cases (9.52%) of adenocarcinoma in situ, 41 cases (48.81%) of minimally invasive adenocarcinoma, and 21 cases (25.00%) of invasive adenocarcinoma. There was no statistical difference between the two groups (p=0.202).

In the hook-wire group, 62 patients (83.78%) underwent anatomic segmentectomy and 12 patients (16.22%) underwent wedge resection. In the medical glue group, 75 patients (89.29%) underwent anatomic segmentectomy, and 9 patients (10.71%) underwent wedge resection. There was no statistical difference between the two groups (p=0.309). All nodules were successfully localized, and VATS resection was completed in all the patients (**Table 1**).

**Table 1:** Comparison of Variables between the 2 groups.

Variables	Hookwire group (n=74)	medical glue group (n=84)	*p*
Gender (Male/Female)	38/36	35/49	0.22
Age (years)	51±11.56	48.93±14.04	0.10
CT nodule size (mm)	9.4±3.0	10.0±3.9	0.48
**Localization**			
Nodule distance to pleural surface (mm)	20.6±13.8	18.0±12.3	0.71
Localization depth within the lung parenchyma (mm)	29.4±13.3	18.5±12.9	0.50
Localization duration (min)	11±3.54	15.0±3.83	0.65
Localization-to-surgery duration (h)	2.12±1.36	16.02±6.59	**0.000**
**Nodule location**			0.325
RUL	30(40.54%)	29(34.52%)	
RML	7(9.46%)	3(3.57%)	
RLL	16(21.62%)	27(32.14%)	
LUL	14(18.92%)	19(22.62%)	
LLL	7(9.46%)	6(7.14%)	
**Complication of localization**			
Minor pneumothorax	26(37.8%)	10(11.9%)	**0.01**
Mild pulmonary hemorrhage	18(24.3%)	11(13.1%)	**0.000**
visual analog scale	5.66±1.86	2.11±1.47	**0.02**
Localization failure	2(2.7%)	0	
**Histopathologic results**			0.202
Benign lesions	3(4.05%)	10(11.90%)	
Atypical adenomatous hyperplasia	3(4.05%)	4(4.76%)	
Adenocarcinoma in situ	3(4.05%)	8(9.52%)	
Minimally invasive adenocarcinoma	46(62.16%)	41(48.81%)	
Invasive adenocarcinoma	19(25.68%)	21(25.00%)	
**Surgical type**			
Wedge	12(16.22%)	9(10.71%)	0.309
Segmentectomy	62(83.78%)	75(89.29%)	

### Statistical difference in the variables between the two groups

The average time from the end of localization to the start of surgery in the hook-wire group was 2.12 ± 1.36 hours (range: 0.5-7 hours), and the average time from the end of localization to the start of surgery in the medical glue group was 16.02 ± 6.59 hours (range: 1-35 hours) and there was a statistical difference between the two groups (p=0.000). After the localization of pulmonary nodules, a CT scan showed 26 cases of minor pneumothorax (37.8%) in the hook-wire group and 10 cases (11.9%) in the medical glue group, indicating a statistical difference between the two groups (*p*=0.01). Although both groups of patients had minor pneumothorax, they had no obvious symptoms of chest tightness or discomfort, and no chest tube insertion was required for drainage. CT scans in the hook-wire group showed mild pulmonary parenchymal hemorrhage in 18 cases (24.3%) and 11 cases (13.1%) in the medical glue group, indicating a statistically significant difference between the two groups (*p*=0.000). However, neither group of patients showed any serious clinical consequences. Moreover, no hemothorax or air embolism was reported in either group of patients. Two patients (2.7%) in the hook-wire group had hook-wire dislodgement, and the localization success rate was 97.3%. The mean VAS scores of patients in the hook-wire group after localization was 5.66 ± 1.86 (range: 2-10), and the mean VAS scores of patients in the medical glue group after localization was 2.11 ± 1.47 (range: 0-5), resulting in a statistical difference between the two groups (*p*=0.02) (**Table 1**).

### Spearman correlation analysis

Lung parenchyma hemorrhage was significantly correlated with the length of the hook-wire localization needle and the depth of medical glue in the lung parenchyma in both groups (*p*=0.009 and 0.001, respectively). However, there was no significant correlation between the occurrence of pneumothorax and the length of the hook-wire localization needle, or the depth of medical glue in the lung parenchyma (*p*=0.959 and 0.205, respectively) (**Table 2**).

**Table 2:** Results of Spearman correlation analysis.

Variables		Length of hook-wire in lung parenchyma (mm)	Medical glue depth within the lung parenchyma (mm)
Minor pneumothorax	*p*-value	0.959	0.205
Mild pulmonary hemorrhage	*p*-value	**0.009**	**0.001**

## Discussion

Lung cancer is the most common type of cancer and is responsible for the highest number of mortalities worldwide (19). The widespread use of low-dose CT has permitted an increasing number of pulmonary nodules (especially ground-glass opacity; GGO) to be detected at an early stage. There is a direct relation between pulmonary nodules and early lung cancer. Up to 50% of lung nodules detected on computed tomography (CT) are malignant, and large-scale randomized clinical trials have also demonstrated that low-dose CT screening reduces lung cancer mortality (20, 21). VATS has been widely used for the diagnosis and treatment of pulmonary nodules and compared with standard thoracotomy, it has fewer complications, shorter hospital stays, and higher patient acceptance (22). Unfortunately, these pulmonary nodules are often invisible on thoracoscopy and cannot be easily palpated. Therefore, it presents a challenge to the surgeon (23), and pre-operative localization is crucial during thoracoscopic surgery (24).

Currently, a variety of pre-operative localization methods for pulmonary nodules has been reported, yet no ideal method has been determined (25). The quick spread of the dye to the pleural surface complicates the use of methylene blue and makes it challenging to detect lung lesions. Pain may also result from dye-induced pleural irritation (26, 27). Intra-operative ultrasonography requires specialized equipment. The resolution of ultrasound is also significantly reduced in patients with asthma, bullae, diffuse emphysema, or pulmonary fibrosis, due to insufficient lung tissue collapse and the presence of lung emphysema. However, since there is no lung parenchymal damage, the risk of pneumothorax and hemoptysis is eliminated (8).

The widespread usage of the hook-wire localization technique benefits from its accurate localization, high success rate, simple operation, fewer complications, and no interference with pathological diagnosis. Alternatively, medical glue may be used for pre-operative localization of pulmonary nodules (11). Such medical glue is made of synthetic cyanoacrylate, which is non-toxic and safe. The glue rapidly polymerizes at ambient temperature in response to anions in bodily fluids to create hard nodules within lung tissue while remaining non-toxic to neighboring tissues. In this study, pulmonary nodules were successfully resected in both groups of patients, and segmentectomy accounted for a higher proportion of the surgical methods, exceeding 80%. All these are inseparable from the successful localization of pulmonary nodules before surgery. The localization rate approaches 100% because the medical adhesive creates hard nodules in the lungs that are easy to touch. Concerning the hook-wire methods, there have been reports of instances when a hook-wire localization needle was found to be displaced or even fall off during the operation (28, 29). Approximately 2.5-13% of pulmonary nodules located by hook-wire may be displaced or even decoupled (30, 31). In our previous study, the hook-wire dislodgement rate was 2.5% (9). In the present study, hook-wire dislodgement occurred in 2 patients (2.7%). First, if the needle is too shallow, the hook-wire may not be sufficiently fixed in the lung tissue. This is particularly common if pneumothorax develops during localization or if the lung tissue collapses after the chest cavity has been opened up during surgery. Second, the hook-wire localization needle may dislocate if the patient moves their arms and shoulders too much after localization. Fortunately, we were able to complete the surgical resection of the lesion by determining the location of the pulmonary nodule through the hook-wire puncture point on the lung surface.

In the event of medical glue usage, localization results in the formation of a solid, hard nodule in the lung; this prevents localization failure even in the event of a pneumothorax or severe arm and shoulder movement. The most common complications associated with localization procedures include pneumothorax and pulmonary parenchymal hemorrhage. The glue sets fast enough to effectively block the puncture site and reduce complications such as lung leak and bleeding (11). In the medical glue group, minor pneumothorax and mild pulmonary parenchymal hemorrhage occurred in 11.9% and 13.1% of patients, respectively. In the hook-wire group, the incidence of minor pneumothorax (37.8%) and mild pulmonary parenchymal hemorrhage (27.3%), were higher than those in the medical glue group. It has been reported in the literature that the incidence of pneumothorax during localization of pulmonary nodules using medical glue was 31.9% compared to 51.4% during hook-wire localization. In addition, pulmonary parenchymal hemorrhage occurred in 13.9% of lung nodules using medical glue, compared with 13.5% with hook-wire placement (32). It can be seen that medical glue has the advantage of reducing pneumothorax and pulmonary hemorrhage during the process of locating pulmonary nodules. Massive air embolism is a rare complication that has been sporadically described in case reports with an incidence of 0.6% (27). In this study, no air embolism was reported in either group of patients.

Studies have shown that the length of hook-wire localization needles in the lung parenchyma is significantly associated with the occurrence of mild pulmonary parenchymal hemorrhage, although no correlation has been shown with the depth of pulmonary nodules (9). In the present study, we found that pulmonary parenchyma hemorrhage was significantly correlated with the length of the hook-wire localization needle in the lung parenchyma and the depth of the medical glue in the lung parenchyma. This was not the case for pneumothorax. Understandably, the deeper the location depth, the greater the chance of encountering blood vessels in the lung tissue and the greater the chance of bleeding.

Medical glue has shown to be more adaptable in the timing of pre-operative localization of pulmonary nodules. The average time from the end of localization to the beginning of the operation in the medical glue group in our study was 16.02 ± 6.59 hours, which was significantly longer than that in the hook-wire duration of 2.12 ± 1.36 hours. So that the thoracic surgeon has more time to plan the procedure for the patient, the medical glue group’s operation time may be chosen up to 35 hours after localization. It has been reported in the literature that the use of hydrogel plugs to locate pulmonary nodules may delay surgery by up to 60 days (33). This is mostly because patients have less discomfort during localization using medical glue, and many do not even experience pain following localization. In addition, the medical glue can remain within the body for a long time without any detrimental consequences. In our study, it was discovered that patients in the medical glue group had lower mean VAS ratings than those in the hook-wire group.

Our research has apparent flaws owing to its lack of randomization and the fact that it is a retrospective analysis conducted at a single location. Furthermore, the sample size of this study is relatively small, which may have a certain impact on the conclusions regarding the safety and efficacy of both types of pre-operative localization.

## Conclusions

Our study found that both types of pre-operative localization methods are safe and effective. However, compared with hook-wire localization, medical glue has fewer complications, is more acceptable to patients, and allows for greater flexibility in the arrangement of the scheduling of surgery.

## Data availability statement

The original contributions presented in the study are included in the article/supplementary material. Further inquiries can be directed to the corresponding authors.

## Ethics statement

The studies involving human participants were reviewed and approved by Huashan Hospital Institutional Review Board of Fudan University. The patients/participants provided their written informed consent to participate in this study. Written informed consent was obtained from the individual(s) for the publication of any potentially identifiable images or data included in this article.

## Author contributions

All the authors conceived and designed the experiments. HZ and XC conducted experiments. YL and ZH prepared the figures and tables. HZ analyzed the data and wrote the manuscript. All the authors reviewed and revised the manuscript. All authors read and approved the manuscript.

## Funding

The study was supported through grants from the National Natural Science Foundation of China, Grant/Award Number: 81602545; Scientific Research Project of Huashan Hospital, Fudan University, Grant/Award Number:2016Q018; and Scientific Research Project of Huashan North Hospital, Fudan University, Grant/Award Number:2015115.

## Conflict of interest

The authors declare that the research was conducted in the absence of any commercial or financial relationships that could be construed as a potential conflict of interest.

## Publisher’s note

All claims expressed in this article are solely those of the authors and do not necessarily represent those of their affiliated organizations, or those of the publisher, the editors and the reviewers. Any product that may be evaluated in this article, or claim that may be made by its manufacturer, is not guaranteed or endorsed by the publisher.
